# Leveraging Deep Learning for IoT Transceiver Identification

**DOI:** 10.3390/e25081191

**Published:** 2023-08-10

**Authors:** Jiayao Gao, Hongfei Fan, Yumei Zhao, Yang Shi

**Affiliations:** 1 School of Software Engineering, Tongji University, Shanghai 200092, China; silentgao91@gmail.com (J.G.);; 2 School of Computer Science and Engineering, The University of New South Wales, Sydney 2052, Australia; 3 Shanghai Pudong Thunisoft Information Technology Corporation Limited, Shanghai 261031, China

**Keywords:** Internet of Things, identification, fingerprinting, deep learning

## Abstract

With the increasing demand for Internet of Things (IoT) network applications, the lack of adequate identification and authentication has become a significant security concern. Radio frequency fingerprinting techniques, which utilize regular radio traffic as the identification source, were then proposed to provide a more secured identification approach compared to traditional security methods. Such solutions take hardware-level characteristics as device fingerprints to mitigate the risk of pre-shared key leakage and lower computational complexity. However, the existing studies suffer from problems such as location dependence. In this study, we have proposed a novel scheme for further exploiting the spectrogram and the carrier frequency offset (CFO) as identification sources. A convolutional neural network (CNN) is chosen as the classifier. The scheme addressed the location-dependence problem in the existing identification schemes. Experimental evaluations with data collected in the real world have indicated that the proposed approach can achieve 80% accuracy even if the training and testing data are collected on different days and at different locations, which is 13% higher than state-of-the-art approaches.

## 1. Introduction

The Internet of Things (IoT) is among the most rapidly growing technologies in recent decades. It is a network consisting of Internet-linked devices and/or sensors that collect and share data without human interaction. Agriculture, smart buildings, manufacturing, and health care are all among the typical IoT application scenarios. IoT makes it feasible to collect and share different kinds of information automatically with the help of IoT sensor networks. During the past three years, the COVID-19 pandemic has also led to the acceleration of IoT technical development, as it helps to reduce direct contact among people, as well as assisting in various ways (e.g., monitoring patients and collecting/transferring data in quarantine areas) [1,2].

With the booming of IoT, much sensitive data (e.g., manufacture monitoring data and medical-related data) are now being transferred via these resource-constrained devices. For example, a Stanford Medicine report shows that more than 2314 exabytes of data had been generated within the medical industry alone by 2020 [3]. However, most of them are transmitted within plaintext networks or simply protected by pre-shared keys that can be easily compromised. As a result, the security of IoT networks has become a significant concern. Traditional security mechanisms like asymmetric-key cryptography (e.g., RSA cryptosystem) have high computational requirements and reliance on trusted third parties, which are unsuitable for resource-limited IoT devices.

Physical-layer security (PLS) is an emerging option to fulfill the security requirements of IoT. It is a powerful technology especially suitable for IoT networks as it commonly supports secure communications with a lower computational power requirement. These PLS-based approaches commonly involve signal processing, physical layer key generation, and physical layer identification [4].

Radio frequency fingerprinting is one of the typical PLS approaches. It utilizes one or several characteristics, including hardware imperfections of the device, as the ‘fingerprint’ to identify specific radio transmitters [5]. Channel-specific features and transmitter-specific features are two major categories of fingerprint features. The former are commonly based on the wireless channel, such as the link signature solution [6], and the latter are device imperfections like carrier frequency offset (CFO) [7]. Such ‘imperfect fingerprints’ either depend on the real-time channel status or are introduced during the chip manufacturing process, both of which are difficult to imitate.

However, many radio frequency fingerprinting studies only focus on WiFi or other wide-band communication technologies. The commonly-used features as fingerprint sources in wide-band communication technologies are either unavailable in narrow-band transceivers or have quite different characteristics. This makes device fingerprinting a challenge in IoT networks, especially in low-power wide-area networks (LPWAN) such as Long Range (LoRa) and Zigbee [8,9,10,11]. Some past research attempted to address the problem. Unfortunately, those with high accuracy usually use location-based features, which makes deployment and re-deployment inconvenient (e.g., fingerprint records need to be updated every time after re-deployment).

To address the above challenges, we propose a novel fingerprinting scheme for LoRa transceivers that is based on the spectrogram of the LoRa packet preamble and the CFO. The proposed scheme also takes advantage of a convolutional neural network (CNN). Experimental evaluations in real-world scenarios have indicated that the performance of the proposed scheme is 13% higher than that of state-of-the-art approaches. The proposed scheme can achieve more than 80% identification accuracy even when the device is moved from its original training location and the training and testing data are collected on different days, which implies that there is no need to collect device fingerprints after deployment and re-deployment.

The rest of this paper is organized as follows. First, we present the related work in Section 2. Then, we illustrate our design in Section 3. The performance evaluations are presented in Section 4, and we conclude this paper in Section 5.

## 2. Related Works

In this section, we present a brief introduction and related studies about the PLS, transceiver fingerprinting, LoRa, CNN, and related challenges for LoRa transceiver identification.

### 2.1. Physical-Layer Security

Password-based identification approaches are rampantly used in IoT and wireless sensor networks for their simplicity and low cost. However, they also face scalability, key revocation, and security issues like key leakage when large-scale deployments are needed. Other security mechanisms like asymmetric-key cryptography can offer a better security level, but they also have high computational requirements or rely on trusted third parties, which makes them inapplicable to IoT devices [3].

The radio PLS was then introduced to address the issues mentioned above. PLS can be used in some critical security operations like node authentication and message confidentiality [12]. For example, physical-layer key generation uses channel reciprocity to generate secret keys between a sender and a receiver to ensure the message confidentiality [13,14]. On the other hand, radio frequency fingerprinting uses the offline-recorded hardware features to identify differences between radio transceivers and verify their eligibility.

Here are some unique advantages of PLS: (i) PLS schemes are theoretically secured if they are properly implemented; (ii) PLS implementations are usually lightweight and without too much computational burden, which makes it suitable for IoT or latency-constrained scenarios; (iii) PLS can be used to generate dynamic keys based on real-time channel estimation and avoid pre-stored key leakage.

### 2.2. Radio Frequency Fingerprinting

**Figure 1:**
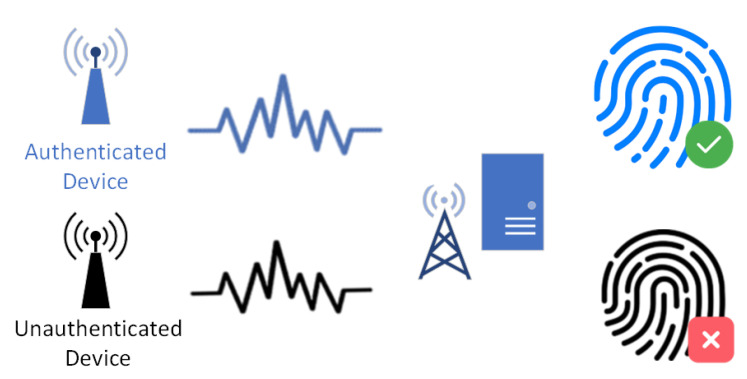
Radio transceiver identification based on device fingerprinting.

As shown in Figure 1, radio frequency fingerprinting is a process that identifies radio transceivers using their characterized ‘fingerprint’ from signal transmission. It is one of the popular methods to enhance wireless communications security. The two main advantages of radio frequency fingerprinting are as follows: (i) it is theoretically secured and hard to imitate, (ii) it is suitable for devices with limited power and computing resources since it does not need extra transmission, but rather uses standard communication signals. Wireless identification is also widely used in localization scenarios [14,15,16,17,18,19,20].

There are two kinds of features that are commonly used as transceiver fingerprints. The first is the channel-specific feature. Typical channel-specific features include the received signal strength [21], link signatures [22,23,24], and the duration of response [25]. However, all these features are location-dependent since they are based on the wireless communication channel. These features are helpful in localization scenarios. However, when coming to authentication, they make redeployment complicated. The second is called the transceiver hardware-specific feature. Unlike channel-specific features based on wireless channels, transceiver hardware-specific features are mainly caused by imperfections in the transceiver chips or different hardware components such as crystal oscillators. Thus, unlike channel-specific features, they are independent of the communication channel. Such features may include clock skews [26], the duration of the packet transient [5,27], and modulation errors. Modulation errors such as the phase error, magnitude error, error vector magnitude, in-phase and quadrature (I/Q) component origin offset, frequency error, SYNC correlation, etc. can also be used for identification [28]. Later, these features were introduced into IoT transceiver identification, such as Zigbee [27] and LoRa [6,29,30].

The proposed scheme will use the hardware-specific features spectrogram and CFO as identification sources. Spectrograms reflect how the signal frequency changes over time. In some research about the voice, spectrograms are commonly considered as voice fingerprints [31]. On the other hand, CFO is a gap between the ideal frequency and the frequency of the transceiver oscillator. It often occurs when the oscillator signal in the receiver does not synchronize with the received signal. According to the CFO, the signal receiver (e.g., a LoRa gateway) can adjust its frequency from the channel center frequency. By executing such an operation, the receiver can achieve carrier lock and be ready for further signal demodulation. We will discuss the calculation in Section 3.4. Since CFO is related to the oscillator, it is an imperfection introduced within the manufacturing process. It is one of the most commonly used transmitter-specific features for transceiver fingerprinting, as the oscillator is widely used in many different kinds of transmitters.

### 2.3. LoRa and Chirp Spread Spectrum

LoRa [32,33,34,35,36,37] and LoRaWAN [11,38,39] are among the most popular LPWAN wireless communication network protocols and operate in an unlicensed spectrum. LoRaWAN is standardized by the LoRa Alliance [40] and LoRa provides the physical-layer foundation for LoRaWAN.

Equation (1) shows the relationship of three crucial LoRa physical-layer parameters:

(1) $$Data\;rate=SF\times \frac{BW}{2^{SF}}\times CR.$$

BW denotes the bandwidth, which is the spectrum size of a channel (e.g., 125 or 500 kHz). SF denotes the spreading factor, which is the number of encoded bits per chirp symbol (e.g., an integer from 7 to 12), and CR stands for the coding rate.

As the name implies, the ability for long-range communications is the advantage of the LoRa protocol. According to the LoRa Alliance, the LoRa communication range can be up to five kilometers in urban areas and fifteen kilometers in suburban areas. LoRa uses the chirp spread spectrum (CSS) to achieve long-range wireless communications. It was initially developed for radar and has received much attention from the research community [41,42,43,44,45,46,47,48].

CSS can relieve the multipath fading impact when operating at very low power (e.g., a button battery) by using the entire allocated bandwidth when broadcasting a signal. Due to its relatively low transmission power and robustness to channel noise and radio multipath effects, CSS has been increasingly adopted in data communication applications over the past 20 years.

**Figure 2:**
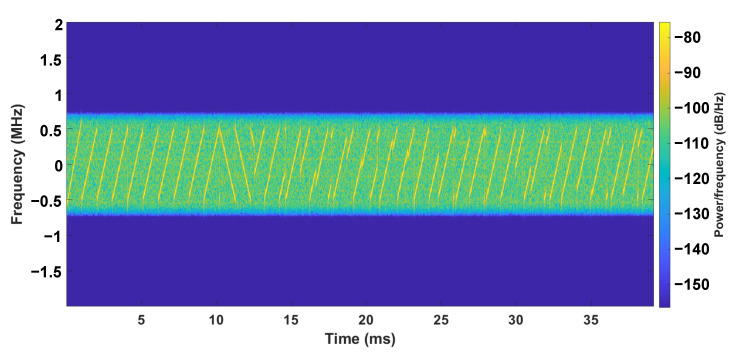
A LoRa packet, consisting of a number of up-chirps and down-chirps. The bandwidth of the first up-chirp is slightly larger than that of the remaining chirps.

Figure 2 shows the signal of a typical LoRa packet preamble. The preamble consists of ten up-chirps and two down-chirps. A standard up-chirp will increase from the lowest frequency of the bandwidth to the highest one. The chirps after the two down-chirps are the payload of the packet. It can be observed that the modulated chirps start from different locations of the bandwidth to encode messages.

### 2.4. Convolutional Neural Network

In recent years, artificial intelligence has become a popular tool in IoT security due to the development of machine learning, including deep learning. Deep learning has been successfully used to learn complex representations of different kinds of data and also as a detector for discriminative features with a high accuracy that is impossible to achieve manually. It has substantially improved the development of image classification, speech, and biometrics recognition [49] compared to previous efforts. Virtual smart assistants [50] and vision for automobiles [51] are some practical examples of deep learning.

Among the different kinds of models and networks, CNN is a class of artificial neural networks that has been widely used in the past several years. The typical application scenarios of CNN include recommendation systems, image classification, natural language processing, etc. A CNN usually consists of multiple building blocks, including convolutional layers, pooling layers, and fully connected layers. Among them, the convolution layer is the core component of CNN. It consists of a stack of mathematical operations and allows CNN to extract relevant patterns from the input data.

Some pioneering studies that combined machine learning with wireless signal processing mainly focused on wide-band systems such as WiFi. For example, support vector machines, a typical machine learning approach, has been used to identify 100 low-cost WiFi transceivers [52], and a generative adaption model has been used for WiFi-based localization [53]. However, very little attention has been paid to applying machine learning to narrow-band radio frequency signal processing. Many features and characteristics in wide-band protocols are unavailable for LoRa, which uses demodulation techniques.

In LoRa, work such as NeLoRa uses deep learning in the demodulation processes [54], while existing LoRa identification approaches apply I/Q samples and spectrum together with CNN, multilayer perceptron, and long short-term memory [30]. As radio frequency fingerprinting can be considered a classification process and the spectrogram is in image format, CNN is chosen in the proposed approach to fulfill the related task.

### 2.5. Limitation of Current Radio Identification Approaches

The feasibility of LoRa identification with deep learning has been explored in [6,55].

DeepLoRa [55] conducted an extensive experiment with 100 transceivers and tried various combinations between different fingerprinting sources and learning models. Its deep learning model may extract CFO and other features from in-phase and quadrature (IQ) samples. It also considered the identification scenarios when testing with a dataset collected on a different day than the training dataset and improved the accuracy by more than 15% using its pioneering data augmentation techniques, but such a problem remains unsolved as its accuracy under such scenarios is not practical enough. Also, the location bias was not discussed.

SLoRa [6] created a feature called linksignature, which can improve the identification accuracy. However, it is location-based, which means the training process needs to be executed after deployment, and an update is also needed every time after the re-deployment of the device. This makes the usability of such solutions a big problem since it is not practical for large-scale IoT networks to update simultaneously.

Other solutions like [30] solve part of the problem, but they did not check the result if the transceivers are moved. It also suffers from its huge training set size.

## 3. System Design

Our LoRa transceiver identification scheme mainly consists of a feature-extraction part and a CNN learning model. The feature extraction is a combination of spectrogram feature processing and CFO calculation. The details are presented in this section.

### 3.1. Identification System in a Nutshell

**Figure 3:**
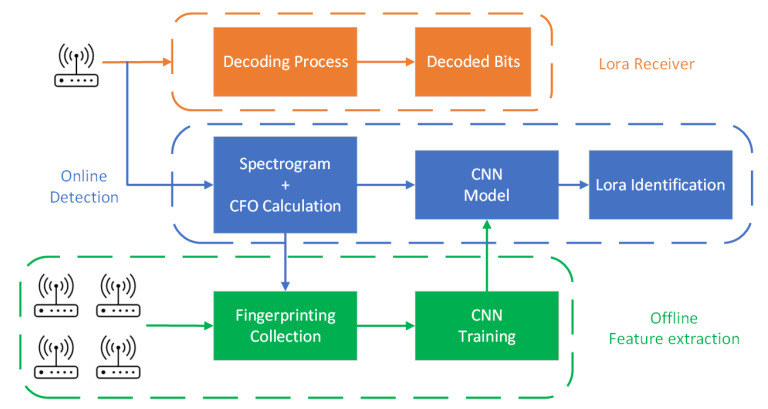
System design.

Figure 3 shows the overall design of our LoRa transceiver identification scheme, which mainly consists of two parts: the offline feature-extraction module (at the bottom of the figure) and the online detection module (in the middle of the figure). The offline module collects signals from legitimate LoRa transceivers. Then, it extracts spectrogram features and CFO according to the feature-extraction algorithms introduced in Section 3.2 and Section 3.4. The feature-extraction results and transceiver labels are sent into the CNN model for training.

When a new LoRa transceiver attempts to join the network, the online module records the signals transmitted from this node and extracts its spectrogram features and CFO. Then, we use the trained CNN model from the offline module to identify if it is a legitimate transceiver and which transceiver it is. It is worth noting that, unlike some existing systems that require sending a signal that carries specific data, our scheme can use radio signals for regular IoT application data exchange to reduce energy consumption since we only use the preamble part of the signal.

### 3.2. LoRa Spectrogram Processing

The spectrogram shows how the spectrum frequencies of a signal change with time. It is usually depicted as a heat map [56]. As described in Section 2, LoRa uses CSS as its modulation method, and thus LoRa spectrograms consist of chirps. Specifically, the preamble part will be the same for every packet, with ten up-chirps and two down-chirps, making it a suitable source for fingerprinting. Figure 2 shows an example of a LoRa packet preamble spectrogram. By employing the spectrogram, we are turning the problem into an image classification problem, which are well-studied by the computer vision community.

The calculation of the spectrogram is based on the I/Q components. They are two sinusoids with the same frequency and are $90^{\circ }$ out of phase. Once the software-defined radio (SDR) receives a signal, it actually stores the signal as a set of I/Q samples. To obtain the spectrogram, a short-time Fourier transform (STFT) is performed according to Equation (2). STFT has been extensively used to analyze non-stationary signals.

(2) $$STFT\left(m,\omega \right)=\sum_{n=-\infty }^{\infty }x\left[n\right]w\left[n-m\right]e^{-j\omega n}.$$

where *m* is the column index of the result, ω is the frequency, x[n] is the signal to be analysed, and w[n] is the window function of length.

The spectrogram can then be calculated based on the STFT result as Equation (3):

(3) $$Sp\left(m,\omega \right)={\left|STFT(m,\omega )\right|}^{2}$$

### 3.3. Singular Value Decomposition

The spectrogram is a matrix of pixels, while the CFO is only a value. This leaves a problem of how to combine these two features. We decided to process the spectrogram with the SVD algorithm first and then connect the single CFO value to the end of the SVD array. The advantage of this operation is that it can largely compress the training input size (e.g., from about 150,000 integers for every signal sample to only about 100 integers) and reduce the training time.

SVD is a factorization of a real or complex matrix. It generalizes the eigendecomposition of a square normal matrix with an orthonormal eigenbasis to any m×n matrix. The SVD of an m×n matrix can be calculated as:

(4) $$M=U\Sigma V^{*}$$

where U is an m×m complex unitary matrix, Σ is an m×n rectangular diagonal matrix with non-negative real numbers on the diagonal, and $V^{*}$ is the conjugate transpose of an n×n complex unitary matrix.

### 3.4. CFO Calculation

In LoRa, the CFO is introduced to indicate the difference between the ideal and observed carrier frequency. The low-cost crystal oscillators embedded in LoRa transceivers produce different carrier frequencies due to hardware manufacturing and temperature differences. IoT transceiver chips like SX1272 from Semtech usually have built-in temperature sensors to compensate. Shen et al. [30] conducted an analysis of the CFO. The result shows that the CFO will have some variation due to continuous working, even with a temperature-compensated oscillator. But it can remain in a particular range if carefully calculated. Therefore, CFO may be used for LoRa device identification.

**Figure 4:**
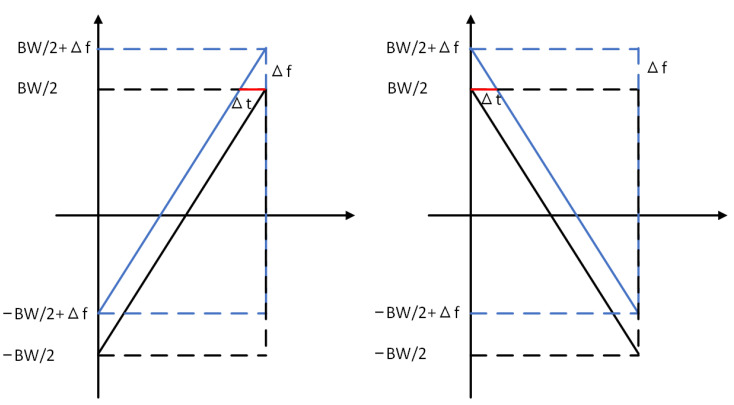
Up-chirp and down-chirp affected by CFO.

**Figure 5:**
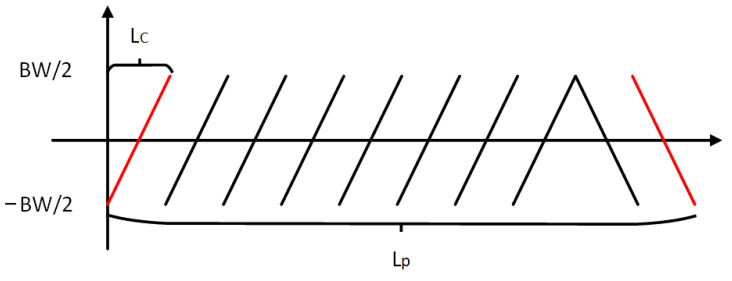
LoRa preamble (up-chirps) and SFD (down-chirps). $L_{p}$ is the number of samples between the cross-correlation peaks of the first up-chirp in the preamble and the last down-chirp in SFD.

CFO can be observed on the receivers (e.g., a gateway). Cross-correlation is used to estimate CFO in some existing methods [57]. If we only observe the frequency range (from −BW/2 to BW/2) as shown in Figure 4, a chirp is shifted in the frequency domain by an offset (Δf). It equals the shift in the time domain (Δt). By correlating the standard up-chirp and down-chirp in the preamble of a packet shown in Figure 5, we can estimate CFO (Δf) from the correlation peaks of the up-chirp and down-chirp. They are shifted either left or right by Δt in Figure 4. However, the accuracy of such estimation methods largely depends on the receiver sampling rate. Even oversampling (coming with more energy and computational resources) cannot guarantee the performance of device identification.

An alternative method is based on the phase of the chirps. For an ideal up-chirp without CFO, its frequency rises from −BW/2 to BW/2 linearly, and its phase is symmetrical. Thus, the beginning and the ending phase of the chirp are the same. If CFO is non-zero (Δf≠0, see Figure 4), the accumulated phase drift Δφ will be:

(5) $$\Delta \varphi =\int_{0}^{T}2\pi \Delta fdt=2\pi T\Delta f,$$

*T* is the length of the chirp, and can be calculated from the BW and the SF as:

(6) $$T=\frac{2^{SF}}{BW}.$$

By substituting *T* in Equation (5) with Equation (6), we can estimate CFO by:

(7) $$\Delta f=\frac{BW}{2\pi \;\cdot \;2^{SF}}\Delta \varphi .$$

Therefore, the phase drift Δφ can be obtained by comparing the phase difference between adjacent up-chirps in the preamble. Below presents the detailed calculation of CFO. However, the phase drift will be bounded modulo 2π between −π to π, making it difficult to estimate those CFO values that are larger than π or smaller than −π. To this end, the CFO will be the sum of a rough estimation of CFO ($CFO_{major}$) and a refining part ($CFO_{minor}$) in the proposed scheme, as:

(8) $$\Delta f=\Delta f_{major}+\Delta f_{minor}$$

The two-step calculation scheme can produce more accurate CFO estimations. Below, we present the detailed calculation of $CFO_{major}$ and $CFO_{minor}$.

$CFO_{major}$ can be calculated by comparing the cross-correlation peaks of the up-chirps and down-chirps in the LoRa packet (see Figure 5). We assume that the CFO is a positive value (Δf>0), as in Figure 4. Thus, the cross-correlation peak is shifted leftwards by Δt. As mentioned above, the accuracy of Δt is limited by the sampling rate (Fs) and the relationship of the measured $\Delta \bar{t}$ and the ground truth Δt is:

(9) $$\Delta \bar{t}=\frac{\left\lfloor \Delta t\;\cdot \;Fs\right\rceil }{Fs}.$$

Since the up-chirp is linear in the frequency domain, the relationship between $\Delta f_{major}$ and $\Delta \bar{t}$ is:

(10) $$\Delta f_{major}=\frac{BW^{2}}{2^{SF}}\;\cdot \;\Delta \bar{t}.$$

Due to the existence of the CFO, the total length of the preamble and start-of-frame delimiter (SFD) (which equals the distance between the cross-correlation peaks of the first up-chirp and the last down-chirp, the red ones in Figure 5) does not match the theoretical length (i.e., 12 times the symbol length). Below, we show the calculation of $\Delta \bar{t}$:

(11) $$\Delta \bar{t}=\frac{1}{2}\;\cdot \;\frac{\left(L_{p}\;mod\;L_{C}\right)}{Fs},$$

where $L_{p}$ is the sample number between the aforementioned two peaks and $L_{C}$ is the sample number in a single chirp, which can be calculated by:

(12) $$L_{C}=\frac{Fs\;\cdot \;2^{SF}}{BW}.$$

Note that Equation (11) is divided by 2, since the Δt appears twice (in both up-chirp and down-chirp). By substituting $\Delta \bar{t}$ in Equation (10), we can obtain $CFO_{major}$ ($\Delta f_{major}$), as:

(13) $$\Delta f_{major}=\frac{BW}{2L_{C}}\left(L_{p}\;mod\;L_{C}\right).$$

Next, we exploit Equation (7) to estimate $CFO_{minor}$.

To estimate the phase drift between two adjacent up-chirps in the preamble, we multiply the first up-chirp with the transpose of the latter one. The average of the phase shift $\Delta \hat{\varphi }$ can be presented as:

(14) $$\Delta \hat{\varphi }=angle\left(\sum_{i=1}^{n}{\vec{c}}_{i}^{H}\;\cdot \;{\vec{c}}_{i+1}\right),$$

where ${\vec{c}}_{i}$ is the first up-chirp (the red one in Figure 5) and *n* represents the number of adjacent up-chirps (e.g., *n* = 7 in Figure 5). With Equation (7), we can obtain the estimation of $CFO_{minor}$ ($\Delta f_{minor}$) as:

(15) $$\Delta f_{minor}=\frac{BW}{2\pi \;\cdot \;2^{SF}}\Delta \hat{\varphi }$$

### 3.5. CNN Model Design

**Figure 6:**
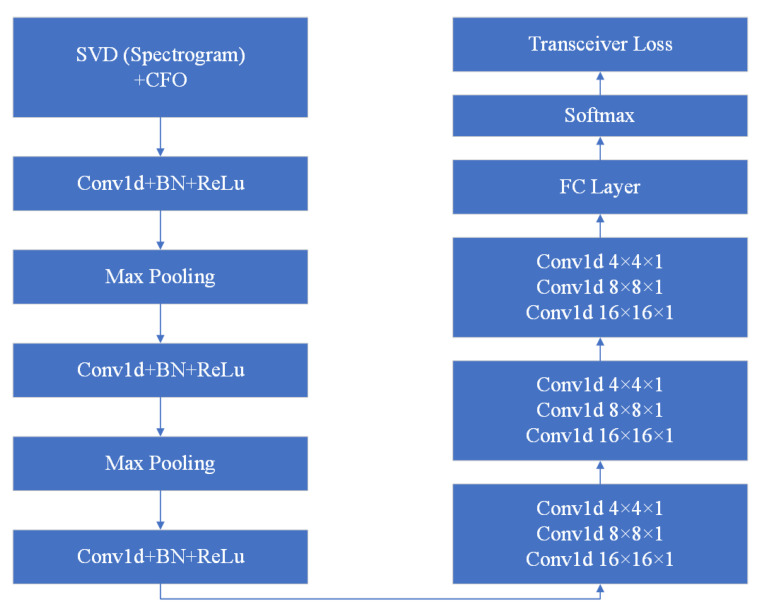
CNN model design.

The proposed scheme uses CNN to extract transceiver features that are widely used in signal processing [30,54]. To extract the features, we use six-layer stacked CNNs shown in Figure 6. In the first three layers of the CNN, a 1D kernel is used as the filter, followed by a batch norm layer and a rectified linear unit. They will normalize the mean and variance of the data and introduce nonlinearity. We also employ a 2 × 2 max-pooling layer to reduce the representation size. In the subsequent three layers of the CNN, each is concatenated with three 1D kernels. The output of the last layer of the feature extractor will then be fed into a fully connected layer for classification. Adam is selected as the optimizer for the training process. Cross entropy is chosen for the loss function. The initial learning rate is 0.0003, and the batch size is 32.

## 4. Evaluation Result

In this section, we will describe our experimental setup and then present the evaluation result based on the data collected in the real world. Our evaluation goals are two-fold: (1) to compare it with state-of-the-art approaches [30] and (2) to evaluate the impact of different system parameters and configurations.

### 4.1. Experimental Setup

**Figure 7:**
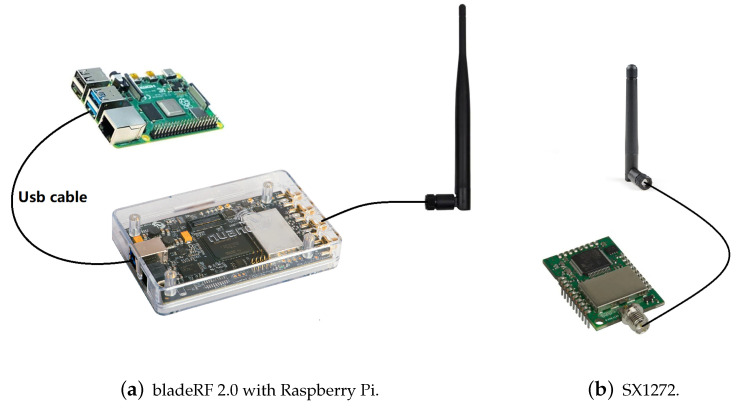
SDR and LoRa nodes.

Our experimental system consists of commercial off-the-shelf (COTS) embedded LoRa nodes to send loRa packets, and a BladeRF 2.0 SDR operates at the 915 MHz band as the receiver. A Raspberry Pi 4 with GnuRadio library installed is connected to the SDR (see Figure 7a). We use the SDR with a single antenna to sample the LoRa radio signal at 2 MHz before recording the analog-to-digital converter (ADC) measurements (samples). Then, the samples are transmitted to the server before being verified by the proposed LoRa transceiver identification scheme. We use COTS MDots with SX1272 transceivers as our embedded LoRa nodes and a 2dbi antenna is connected to Mdot to simulate longer-distance communication (see Figure 7b). Both the BladeRF SDR and MDot specify their LoRa channel bandwidths as 500 kHz.

**Figure 8:**
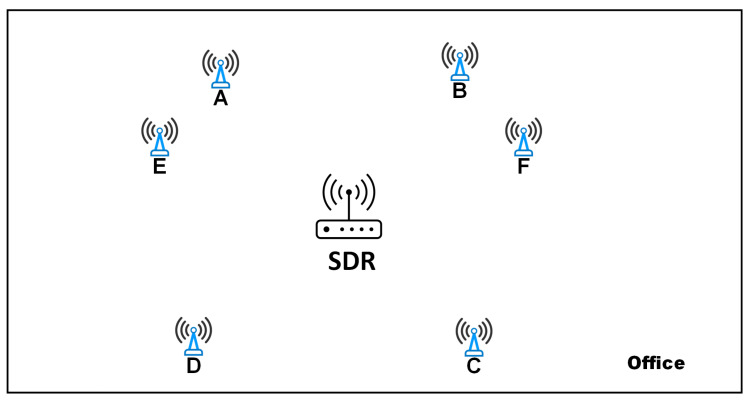
Experimental setup.

The experiment site is an office with some desks and chairs inside. We deploy the embedded LoRa nodes at six different locations (A to F) and place the SDR in the same place. The locations we place MDots are shown in Figure 8. We place MDots at four locations (A to D) on Days 1 and 2 as the training set. On Day 3, we place MDots at two locations (E and F) as the testing set. The distance between each location and the SDR is about three to four meters. We also use a 2dbi antenna to simulate longer-distance communication. Xu et al. [58] have found that due to fading effects, antenna de-tuning, and path-loss, the reception quality can fluctuate heavily, even within 10 m in a line-of-sight scenario. Ten MDots (the same model from the same manufacturer) are involved as our identification targets. All packets use SF=10, and the transmission rate is 3900 bps. All ten MDots are deployed at the same spot (e.g., location A) one by one for a single round of collection and transit two hundred packets at the same maximum power level (i.e., 20 dB).

To avoid bias caused by the environmental temperature, power supply level, or hours of continuous operation, the following methods are also applied to the data-collection process: (i) the experiments are conducted in an air-conditioned office, and thus the environmental temperature remains stable; (ii) the Mdot is powered by an AA battery box, and we use brand new batteries for every collection round, which ensures the stability of the power supply level among the experiments; (iii) each transceiver had rested for at least 4 h before data collection to avoid the impact of continuous operations.

### 4.2. Performance and Discussion

Before presenting the performance, we first illustrate the different training configurations in Table 1.

#### 4.2.1. Comparison with State of the Art

We compare our scheme with the state-of-the-art LoRa radio frequency fingerprint identification approaches of Shen et al. [30]. The state-of-the-art approach is based on the channel-independent spectrogram and a CNN model. We applied both methods to our data set described in Section 4.1 under two different scenarios: (i) the training and testing sets are collected at the same location on the same day and (ii) the training and testing sets are collected at different locations on different days.

**Figure 9:**
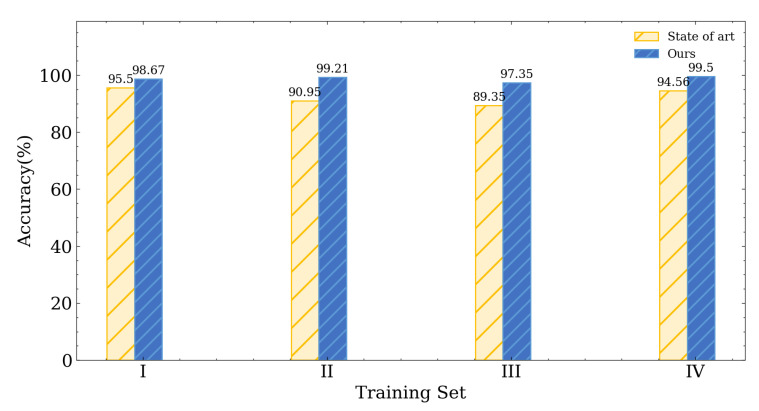
Experimental result with testing set on same day at same location.

**Training and testing sets at the same location on the same day:** In this scenario, we simply separate 20% of the data from the training set as our testing data. Figure 9 shows that our scheme achieves a high accuracy rate ranging from 97.3% to 99.5% when the training and testing sets are collected at the same location on the same day, which surpasses the state-of-the-art approach in all same-day scenarios (training sets I, II, III, and IV).

**Figure 10:**
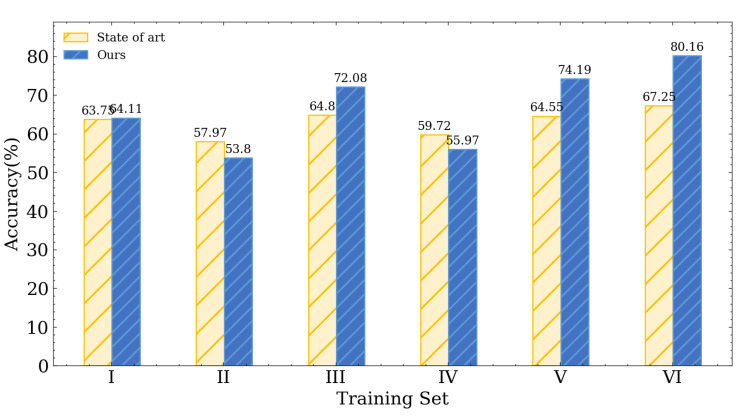
Experimental result with testing set on different day (Day 3) and at different locations (E, F).

**Training and testing sets at different locations on different days:** For this scenario, we utilize the data gathered at locations E and F on day 3 as testing sets, which differ from the training set in terms of location and time. Figure 10 shows that the highest accuracy of the approach proposed in [30] is 67.52%. In contrast, our approach has 80.77%, which outperforms the state-of-the-art LoRa transceiver identification schemes by about 13%.

#### 4.2.2. Impact of Different Training Configurations

We also checked the impact of different training set configurations under different-location different-day scenarios. According to Figure 10, when training with single-day data sets (training sets I, II, III, and IV), the highest accuracy is only about 72%. But when we use two locations on two different days as the training set (training set V), the accuracy then rises to 74.19%. If we further involve four locations on two different days (training set VI), the accuracy can reach 80.16%. Based on our analysis, including a wider range of data from various locations and days in the training set will lead to enhanced identification accuracy in different-location different-day scenarios.

## 5. Conclusions and Discussion

In this study, we have proposed a new identification scheme for LoRa to address the location-dependent challenge in IoT transceiver fingerprinting. The existing identification scheme [30] utilizes channel-related features that are location-dependent. In contrast, our proposed approach utilizes location-independent features (i.e., spectrogram and CFO). The experimental evaluation has indicated that the proposed approach is location-independent and achieves above 80% identification accuracy in the situation where the training and testing data are collected on different days, which outperforms the state-of-the-art approaches by 13%.

The result of our paper provides a promising solution for IoT network authentication. Such a solution can record the device fingerprint before deployment and avoid the trouble of updating the fingerprint record after redeployment. But since it is only based on a LoRa protocol, there may still be some bias related to specific protocols or devices. We may extend the scheme to other IoT protocols in our future work.

## Figures and Tables

**Table 1:** Training configuration.

Training Set	Configuration
I	A, B on Day 1
II	A, B on Day 2
III	A, B, C, D on Day 1
IV	A, B, C, D on Day 2
V	A, B on Day 1 and 2
VI	A, B, C, D on Day 1 and 2

## Data Availability

The data presented in this study are available on request from the corresponding author. The data are not publicly available due to some related research are not published yet.

## Abbreviations

The following abbreviations are used in this manuscript:

IoT	Internet of Things
CFO	carrier frequency offset
CNN	convolutional neural network
SVD	singular value decomposition
LoRa	long range
PLS	physical-layer security
LPWAN	low-power wide-area networks
